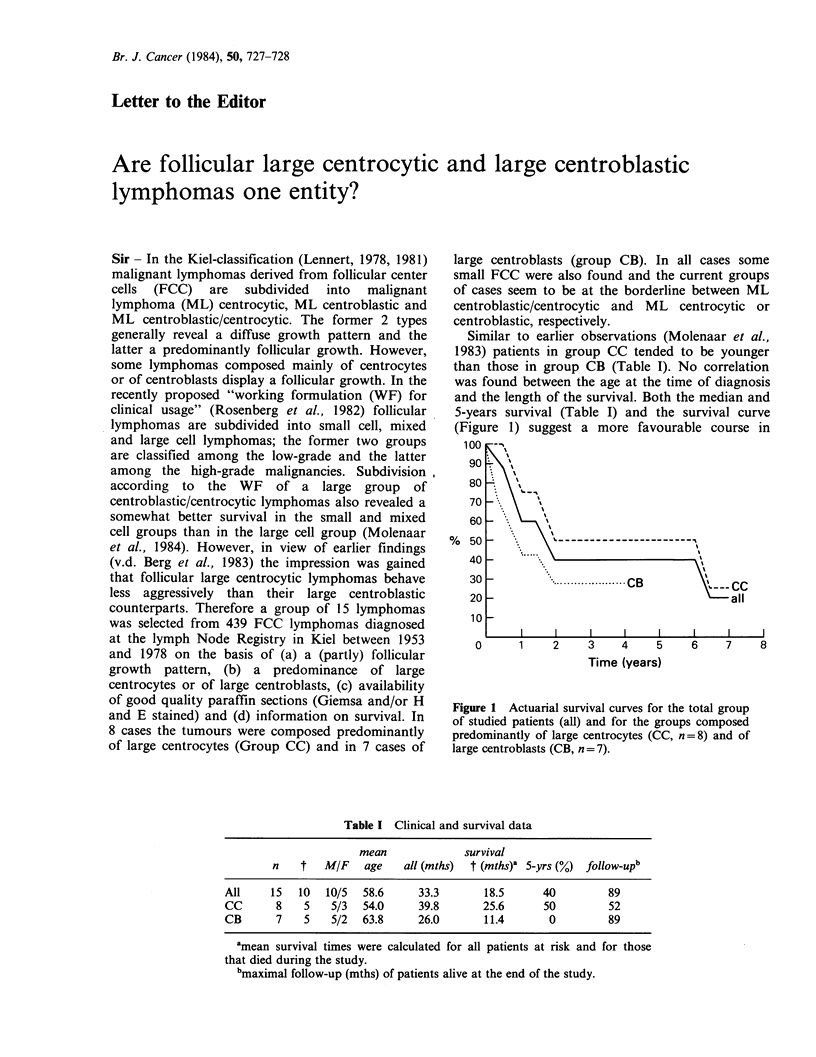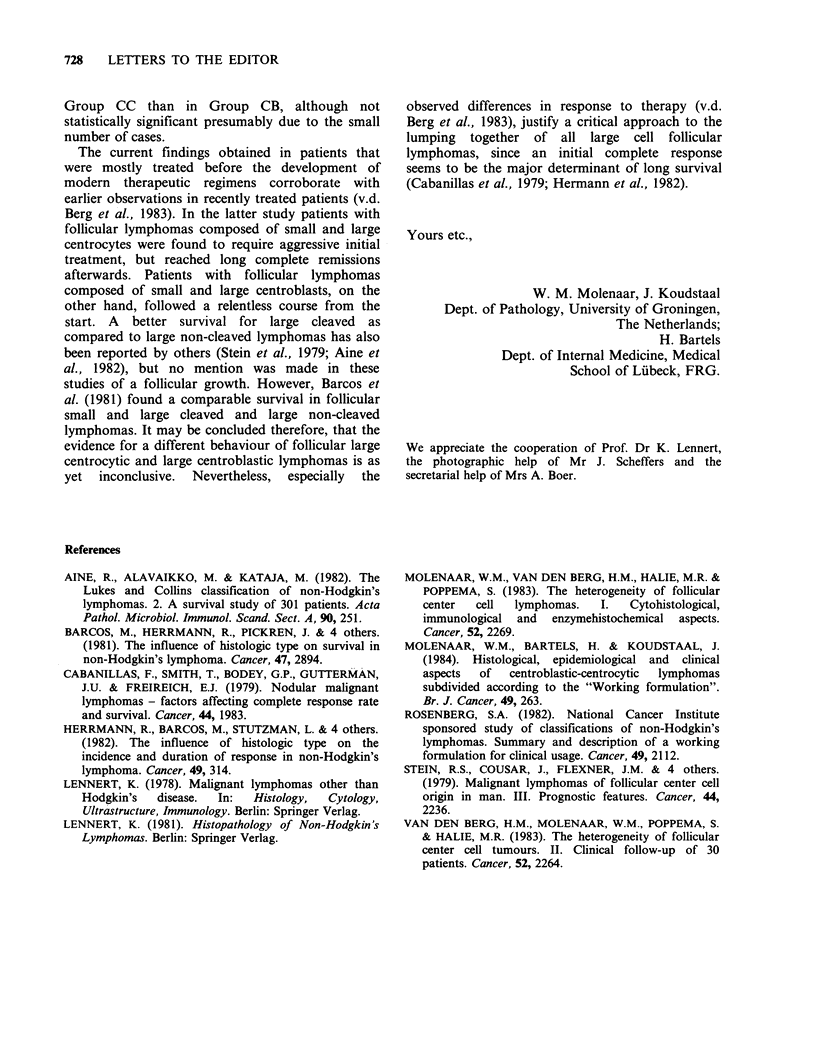# Are follicular large centrocytic and large centroblastic lymphomas one entity?

**DOI:** 10.1038/bjc.1984.245

**Published:** 1984-11

**Authors:** W. M. Molenaar, J. Koudstaal, H. Bartels


*Br. J. Cancer* (1984), **50**, 727–728

### Letter to the Editor

### Are follicular large centrocytic and large centroblastic lymphomas one entity?

**Sir** – In the Kiel-classification (Lennert, 1978, 1981) malignant lymphomas derived from follicular center cells (FCC) are subdivided into malignant lymphoma (ML) centrocytic, ML centroblastic and ML centroblastic/centrocytic. The former 2 types generally reveal a diffuse growth pattern and the latter a predominantly follicular growth. However, some lymphomas composed mainly of centrocytes or of centroblasts display a follicular growth. In the recently proposed "working formulation (WF) for clinical usage" (Rosenberg *et al.*, 1982) follicular lymphomas are subdivided into small cell, mixed and large cell lymphomas; the former two groups are classified among the low-grade and the latter among the high-grade malignancies. Subdivision according to the WF of a large group of centroblastic/centrocytic lymphomas also revealed a somewhat better survival in the small and mixed cell groups than in the large cell group (Molenaar *et al.*, 1984). However, in view of earlier findings (v.d. Berg *et al.*, 1983) the impression was gained that follicular large centrocytic lymphomas behave less aggressively than their large centroblastic counterparts. Therefore a group of 15 lymphomas was selected from 439 FCC lymphomas diagnosed at the lymph Node Registry in Kiel between 1953 and 1978 on the basis of (a) a (partly) follicular growth pattern, (b) a predominance of large centrocytes or of large centroblasts, (c) availability of good quality paraffin sections (Giemsa and/or H and E stained) and (d) information on survival. In 8 cases the tumours were composed predominantly of large centrocytes (Group CC) and in 7 cases of large centroblasts (group CB). In all cases some small FCC were also found and the current groups of cases seem to be at the borderline between ML centroblastic/centrocytic and ML centrocytic or centroblastic, respectively.

Similar to earlier observations (Molenaar *et al.*, 1983) patients in group CC tended to be younger than those in group CB (Table I). No correlation was found between the age at the time of diagnosis and the length of the survival. Both the median and 5-years survival (Table I) and the survival curve (Figure 1) suggest a more favourable course in

**Figure 1** Actuarial survival curves for the total group of studied patients (all) and for the groups composed predominantly of large centrocytes (CC, $n=8$) and of large centroblasts (CB, $n=7$).

**Table I** Clinical and survival data

| | *n* | † | *M/F* | *mean age* | *survival all (mths)* | *survival † (mths)*[a] | *5-yrs (%)* | *follow-up*[b] |
|---|---|---|---|---|---|---|---|---|
| All | 15 | 10 | 10/5 | 58.6 | 33.3 | 18.5 | 40 | 89 |
| CC | 8 | 5 | 5/3 | 54.0 | 39.8 | 25.6 | 50 | 52 |
| CB | 7 | 5 | 5/2 | 63.8 | 26.0 | 11.4 | 0 | 89 |

[a]mean survival times were calculated for all patients at risk and for those that died during the study.
[b]maximal follow-up (mths) of patients alive at the end of the study.

Group CC than in Group CB, although not statistically significant presumably due to the small number of cases.

The current findings obtained in patients that were mostly treated before the development of modern therapeutic regimens corroborate with earlier observations in recently treated patients (v.d. Berg *et al.*, 1983). In the latter study patients with follicular lymphomas composed of small and large centrocytes were found to require aggressive initial treatment, but reached long complete remissions afterwards. Patients with follicular lymphomas composed of small and large centroblasts, on the other hand, followed a relentless course from the start. A better survival for large cleaved as compared to large non-cleaved lymphomas has also been reported by others (Stein *et al.*, 1979; Aine *et al.*, 1982), but no mention was made in these studies of a follicular growth. However, Barcos *et al.* (1981) found a comparable survival in follicular small and large cleaved and large non-cleaved lymphomas. It may be concluded therefore, that the evidence for a different behaviour of follicular large centrocytic and large centroblastic lymphomas is as yet inconclusive. Nevertheless, especially the observed differences in response to therapy (v.d. Berg *et al.*, 1983), justify a critical approach to the lumping together of all large cell follicular lymphomas, since an initial complete response seems to be the major determinant of long survival (Cabanillas *et al.*, 1979; Hermann *et al.*, 1982).

Yours etc.,

W. M. Molenaar, J. Koudstaal
Dept. of Pathology, University of Groningen,
The Netherlands;
H. Bartels
Dept. of Internal Medicine, Medical
School of Lübeck, FRG.

We appreciate the cooperation of Prof. Dr K. Lennert, the photographic help of Mr J. Scheffers and the secretarial help of Mrs A. Boer.

**References**

AINE, R., ALAVAIKKO, M. & KATAJA, M. (1982). The Lukes and Collins classification of non-Hodgkin's lymphomas. 2. A survival study of 301 patients. *Acta Pathol. Microbiol. Immunol. Scand. Sect. A*, **90**, 251.

BARCOS, M., HERRMANN, R., PICKREN, J. & 4 others. (1981). The influence of histologic type on survival in non-Hodgkin's lymphoma. *Cancer*, **47**, 2894.

CABANILLAS, F., SMITH, T., BODEY, G.P., GUTTERMAN, J.U. & FREIREICH, E.J. (1979). Nodular malignant lymphomas – factors affecting complete response rate and survival. *Cancer*, **44**, 1983.

HERRMANN, R., BARCOS, M., STUTZMAN, L. & 4 others. (1982). The influence of histologic type on the incidence and duration of response in non-Hodgkin's lymphoma. *Cancer*, **49**, 314.

LENNERT, K. (1978). Malignant lymphomas other than Hodgkin's disease. In: *Histology, Cytology, Ultrastructure, Immunology*. Berlin: Springer Verlag.

LENNERT, K. (1981). *Histopathology of Non-Hodgkin's Lymphomas*. Berlin: Springer Verlag.

MOLENAAR, W.M., VAN DEN BERG, H.M., HALIE, M.R. & POPPEMA, S. (1983). The heterogeneity of follicular center cell lymphomas. I. Cytohistological, immunological and enzymehistochemical aspects. *Cancer*, **52**, 2269.

MOLENAAR, W.M., BARTELS, H. & KOUDSTAAL, J. (1984). Histological, epidemiological and clinical aspects of centroblastic-centrocytic lymphomas subdivided according to the "Working formulation". *Br. J. Cancer*, **49**, 263.

ROSENBERG, S.A. (1982). National Cancer Institute sponsored study of classifications of non-Hodgkin's lymphomas. Summary and description of a working formulation for clinical usage. *Cancer*, **49**, 2112.

STEIN, R.S., COUSAR, J., FLEXNER, J.M. & 4 others. (1979). Malignant lymphomas of follicular center cell origin in man. III. Prognostic features. *Cancer*, **44**, 2236.

VAN DEN BERG, H.M., MOLENAAR, W.M., POPPEMA, S. & HALIE, M.R. (1983). The heterogeneity of follicular center cell tumours. II. Clinical follow-up of 30 patients. *Cancer*, **52**, 2264.